# Poly[(μ_2_-4,4′-bipyridine-κ^2^ *N*:*N*′)(μ_2_-2,2-dimeth­yl­cyclo­pentane-1,3-dicarboxyl­ato-κ^4^ *O* ^1^,*O* ^1′^:*O* ^3^,*O* ^3′^)cadmium]

**DOI:** 10.1107/S1600536811042656

**Published:** 2011-10-22

**Authors:** Xian-Fa Zhang, Shan Gao, Seik Weng Ng

**Affiliations:** a Key Laboratory of Functional Inorganic Materials Chemistry, Ministry of Education, Heilongjiang University, Harbin 150080, People’s Republic of China; b Department of Chemistry, University of Malaya, 50603 Kuala Lumpur, Malaysia; c Chemistry Department, Faculty of Science, King Abdulaziz University, PO Box 80203 Jeddah, Saudi Arabia

## Abstract

In the title polymeric compound, [Cd(C_9_H_12_O_4_)(C_10_H_8_N_2_)]_*n*_, the Cd^II^ atom is located on a twofold rotation axis and is coordinated by two 4,4′-bipyridine ligands and two 2,2-dimethyl­cyclo­pentane-1,3-dicarboxyl­ate ions. The carboxyl­ate ion and the *N*-heterocycle both function as bridges to link adjacent Cd^II^ atoms to result in the formation of a layer structure parallel to (010). The mid-point of the central C—C bond of the 4,4′-bipyridine ligand is located on an inversion center. In the crystal, the carboxyl­ate ion is disordered over a twofold rotation axis in respect of its methyl group and the cyclo­pentane ring.

## Related literature

For the synthesis of (1*R*,3*S*)-1,2,2-trimethyl­cyclo­pentane-1,3-dicarb­oxy­lic acid, see: Adhya *et al.* (1956 ▶); Camps & Jaime (1981 ▶).
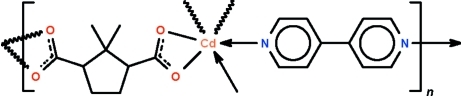


## Experimental

### 

#### Crystal data

[Cd(C_9_H_12_O_4_)(C_10_H_8_N_2_)]
*M* *_r_* = 452.77
Monoclinic, 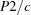

*a* = 9.8527 (5) Å
*b* = 7.2830 (4) Å
*c* = 14.6432 (9) Å
β = 100.879 (1)°
*V* = 1031.87 (10) Å^3^
*Z* = 2
Mo *K*α radiation
μ = 1.08 mm^−1^
*T* = 293 K
0.18 × 0.15 × 0.13 mm

#### Data collection

Rigaku R-AXIS RAPID IP diffractometer
Absorption correction: multi-scan (*ABSCOR*; Higashi, 1995 ▶) *T*_min_ = 0.829, *T*_max_ = 0.872
9640 measured reflections
2364 independent reflections
1774 reflections with *I* > 2σ(*I*)
*R*_int_ = 0.040

#### Refinement

*R*[*F* ^2^ > 2σ(*F* ^2^)] = 0.054
*wR*(*F* ^2^) = 0.171
*S* = 1.12
2364 reflections
150 parameters
51 restraints
H-atom parameters constrained
Δρ_max_ = 1.03 e Å^−3^
Δρ_min_ = −1.27 e Å^−3^

### 

Data collection: *RAPID-AUTO* (Rigaku, 1998 ▶); cell refinement: *RAPID-AUTO*; data reduction: *CrystalClear* (Rigaku/MSC, 2002 ▶); program(s) used to solve structure: *SHELXS97* (Sheldrick, 2008 ▶); program(s) used to refine structure: *SHELXL97* (Sheldrick, 2008 ▶); molecular graphics: *X-SEED* (Barbour, 2001 ▶); software used to prepare material for publication: *publCIF* (Westrip, 2010 ▶).

## Supplementary Material

Crystal structure: contains datablock(s) global, I. DOI: 10.1107/S1600536811042656/xu5349sup1.cif

Structure factors: contains datablock(s) I. DOI: 10.1107/S1600536811042656/xu5349Isup2.hkl

Additional supplementary materials:  crystallographic information; 3D view; checkCIF report

## Figures and Tables

**Table 1:** Selected bond lengths (Å)

Cd1—N1	2.328 (5)
Cd1—O1	2.303 (5)
Cd1—O2	2.359 (5)

**Fig. 1.**
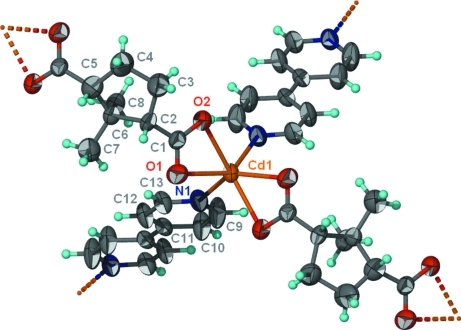
Thermal ellipsoid plot (Barbour, 2001) of a portion of polymeric Cd(C10H8N2)(C9H12O4) at the 50% probability level; hydrogen atoms are drawn as spheres of arbitrary radius. The carboxylate group is disordered over a twofold rotation axis.

## supplementary crystallographic information

### Comment

The compound is the racemic product resulting from the reaction of cadium(II) ions and the deprotonated, optically active (1*R*,3*S*)-1,2,2-trimethylcyclopentane-1,3-dicarboxylate ion. The 1-methyl group is cleaved under hydrothermal conditions to result in the formation of polymeric Cd(C_10_H_8_N_2_)(C_9_H_12_O_4_) (Scheme I), which is racemic. The carboxylate ion and the *N*-heterocycle both function as bridges to link adjacent six-coordinate Cd^II^ atoms to result in the formation of a layer structure (Fig. 1). Racemic 2,2-dimethylcyclopentane-1,3-dicarboxylic acid is not known in the chemical literature; (1*R*,3*S*)-,2,2-trimethylcyclopentane-1,3-dicarboxylic acid is known as apocamphoric acid; its synthesis involves several steps (Adhya *et al.*, 1956; Camps & Jaime, 1981).

### Experimental

Cadmium nitrate (1 mmol), (+)-camphoric acid (1 mmol), 4,4'-bipyridine (1 mmol) and sodium hydroxide (2 mmol) were mixed in water (8 ml). The mixture was placed in a 23-ml, Teflon-lined, stainless-steel Parr bomb. This was heated at 413 K for 3 days. Colorless crystals were isolated when the bomb was cooled slowly to room temperature.

### Refinement

Carbon-bound H-atoms were placed in calculated positions (C–H 0.93–0.96 Å) and were included in the refinement in the riding model approximation, with *U*(H) set to 1.2–1.5*U*(C).

The 2,2-dimethylcyclopentadicarboxylate dianion is disordered over a twofold rotation axis in respect of the methyl groups and the cyclopentane ring; the carboxyl –CO_2_ unit is ordered. In the disordered part, all carbon-carbon distances were restrained to 1.50 ± 0.01 Å; the anisotropic temperature factors were restrained to be nearly isotropic.

The final difference Fourier map had a peak in the vicinity of H4a and a hole in the vicinity of Cd1.

The temperature factors of the two oxygen atoms are large, but are not significantly larger than that of the carbon atom to which they are connected. The temperature factors of the carbon atoms of the pyridine ring are also somewhat large; splitting the ring as two overlapping rings in a disorder model did not improve the refinement much.

### Crystal data

**Table:**

[Cd(C_9_H_12_O_4_)(C_10_H_8_N_2_)]	*F*(000) = 456
*M**_r_* = 452.77	*D*_x_ = 1.457 Mg m^−^^3^
Monoclinic, *P*2/*c*	Mo *K*α radiation, λ = 0.71073 Å
Hall symbol: -P 2yc	Cell parameters from 7550 reflections
*a* = 9.8527 (5) Å	θ = 3.1–27.4°
*b* = 7.2830 (4) Å	µ = 1.08 mm^−^^1^
*c* = 14.6432 (9) Å	*T* = 293 K
β = 100.879 (1)°	Prism, colorless
*V* = 1031.87 (10) Å^3^	0.18 × 0.15 × 0.13 mm
*Z* = 2	

### Data collection

**Table:**

Rigaku R-AXIS RAPID IP diffractometer	2364 independent reflections
Radiation source: fine-focus sealed tube	1774 reflections with *I* > 2σ(*I*)
graphite	*R*_int_ = 0.040
ω scans	θ_max_ = 27.4°, θ_min_ = 3.1°
Absorption correction: multi-scan (*ABSCOR*; Higashi, 1995)	*h* = −12→12
*T*_min_ = 0.829, *T*_max_ = 0.872	*k* = −9→8
9640 measured reflections	*l* = −18→18

### Refinement

**Table:**

Refinement on *F*^2^	Primary atom site location: structure-invariant direct methods
Least-squares matrix: full	Secondary atom site location: difference Fourier map
*R*[*F*^2^ > 2σ(*F*^2^)] = 0.054	Hydrogen site location: inferred from neighbouring sites
*wR*(*F*^2^) = 0.171	H-atom parameters constrained
*S* = 1.12	*w* = 1/[σ^2^(*F*_o_^2^) + (0.0794*P*)^2^ + 2.2957*P*] where *P* = (*F*_o_^2^ + 2*F*_c_^2^)/3
2364 reflections	(Δ/σ)_max_ = 0.001
150 parameters	Δρ_max_ = 1.03 e Å^−^^3^
51 restraints	Δρ_min_ = −1.27 e Å^−^^3^

### Fractional atomic coordinates and isotropic or equivalent isotropic displacement parameters (Å^2^)

**Table:**

	*x*	*y*	*z*	*U*_iso_*/*U*_eq_	Occ. (<1)
Cd1	0.5000	0.62834 (9)	0.2500	0.0559 (3)	
O1	0.3446 (5)	0.7854 (9)	0.3202 (4)	0.0823 (15)	
O2	0.2784 (5)	0.7239 (10)	0.1742 (3)	0.0863 (16)	
N1	0.5040 (6)	0.3945 (8)	0.3589 (4)	0.0662 (14)	
C1	0.2552 (5)	0.7942 (8)	0.2473 (4)	0.0550 (13)	
C2	0.1213 (9)	0.8972 (19)	0.2457 (10)	0.056 (9)	0.50
H2	0.1436	1.0054	0.2855	0.067*	0.50
C3	0.0477 (13)	0.965 (3)	0.1520 (10)	0.089 (4)	0.50
H3A	0.0618	0.8805	0.1034	0.107*	0.50
H3B	0.0825	1.0848	0.1388	0.107*	0.50
C4	−0.1058 (14)	0.977 (3)	0.1565 (12)	0.105 (5)	0.50
H4A	−0.1358	1.1040	0.1544	0.126*	0.50
H4B	−0.1609	0.9116	0.1047	0.126*	0.50
C5	−0.1204 (10)	0.889 (3)	0.2478 (13)	0.080 (14)	0.50
H5	−0.1183	0.9937	0.2902	0.096*	0.50
C6	0.0133 (11)	0.7874 (13)	0.2848 (7)	0.055 (3)	0.50
C7	0.0441 (18)	0.764 (3)	0.3889 (8)	0.093 (5)	0.50
H7A	0.0492	0.8828	0.4181	0.140*	0.50
H7B	0.1307	0.7016	0.4072	0.140*	0.50
H7C	−0.0282	0.6937	0.4078	0.140*	0.50
C8	0.009 (5)	0.5890 (13)	0.261 (2)	0.067 (5)	0.50
H8A	−0.0122	0.5751	0.1944	0.100*	0.50
H8B	−0.0599	0.5288	0.2880	0.100*	0.50
H8C	0.0980	0.5348	0.2843	0.100*	0.50
C9	0.5964 (9)	0.2616 (14)	0.3637 (7)	0.103 (3)	
H9	0.6640	0.2744	0.3275	0.124*	
C10	0.6011 (9)	0.1056 (12)	0.4177 (7)	0.090 (3)	
H10	0.6697	0.0179	0.4178	0.109*	
C11	0.5015 (7)	0.0832 (9)	0.4713 (4)	0.0557 (13)	
C12	0.4087 (8)	0.2236 (11)	0.4689 (5)	0.076 (2)	
H12	0.3419	0.2171	0.5060	0.091*	
C13	0.4122 (8)	0.3746 (10)	0.4125 (5)	0.0710 (18)	
H13	0.3465	0.4663	0.4125	0.085*	

### Atomic displacement parameters (Å^2^)

**Table:**

	*U*^11^	*U*^22^	*U*^33^	*U*^12^	*U*^13^	*U*^23^
Cd1	0.0517 (4)	0.0662 (4)	0.0532 (4)	0.000	0.0187 (2)	0.000
O1	0.061 (3)	0.110 (4)	0.074 (3)	0.018 (3)	0.008 (2)	−0.020 (3)
O2	0.056 (2)	0.140 (5)	0.065 (3)	0.005 (3)	0.016 (2)	−0.014 (3)
N1	0.072 (3)	0.069 (3)	0.062 (3)	0.007 (3)	0.025 (3)	0.013 (2)
C1	0.046 (3)	0.059 (3)	0.060 (3)	−0.004 (3)	0.012 (2)	0.005 (3)
C2	0.040 (10)	0.058 (11)	0.071 (11)	−0.010 (6)	0.015 (6)	−0.001 (6)
C3	0.075 (7)	0.105 (8)	0.088 (8)	0.012 (7)	0.017 (6)	0.028 (7)
C4	0.087 (8)	0.126 (9)	0.100 (9)	−0.030 (7)	0.017 (7)	0.040 (8)
C5	0.070 (16)	0.080 (16)	0.089 (15)	0.005 (8)	0.016 (9)	0.000 (8)
C6	0.049 (5)	0.060 (5)	0.056 (5)	−0.006 (5)	0.013 (5)	0.001 (4)
C7	0.086 (8)	0.110 (9)	0.086 (8)	0.007 (7)	0.021 (7)	0.005 (7)
C8	0.058 (9)	0.065 (5)	0.079 (11)	−0.002 (8)	0.018 (8)	0.003 (7)
C9	0.094 (6)	0.113 (7)	0.121 (7)	0.026 (5)	0.066 (5)	0.055 (6)
C10	0.084 (5)	0.095 (6)	0.106 (6)	0.027 (4)	0.052 (5)	0.040 (5)
C11	0.064 (3)	0.062 (3)	0.043 (3)	−0.002 (3)	0.015 (2)	−0.003 (2)
C12	0.090 (5)	0.079 (4)	0.071 (4)	0.015 (4)	0.046 (4)	0.015 (4)
C13	0.082 (4)	0.074 (4)	0.063 (4)	0.011 (4)	0.030 (3)	0.007 (3)

### Geometric parameters (Å, °)

**Table:**

Cd1—N1^i^	2.328 (5)	C4—H4B	0.9700
Cd1—N1	2.328 (5)	C5—C1^ii^	1.512 (9)
Cd1—O1	2.303 (5)	C5—C6	1.520 (10)
Cd1—O1^i^	2.303 (5)	C5—H5	0.9800
Cd1—O2^i^	2.359 (5)	C6—C8	1.487 (9)
Cd1—O2	2.359 (5)	C6—C7	1.507 (9)
Cd1—C1^i^	2.691 (5)	C7—H7A	0.9600
O1—C1	1.250 (7)	C7—H7B	0.9600
O2—C1	1.247 (7)	C7—H7C	0.9600
N1—C13	1.313 (9)	C8—H8A	0.9600
N1—C9	1.322 (10)	C8—H8B	0.9600
C1—C5^ii^	1.512 (9)	C8—H8C	0.9600
C1—C2	1.514 (9)	C9—C10	1.380 (11)
C2—C3	1.509 (10)	C9—H9	0.9300
C2—C6	1.526 (9)	C10—C11	1.377 (9)
C2—H2	0.9800	C10—H10	0.9300
C3—C4	1.528 (10)	C11—C12	1.368 (10)
C3—H3A	0.9700	C11—C11^iii^	1.478 (12)
C3—H3B	0.9700	C12—C13	1.379 (10)
C4—C5	1.513 (10)	C12—H12	0.9300
C4—H4A	0.9700	C13—H13	0.9300
			
O1—Cd1—O1^i^	120.4 (3)	C5—C4—H4A	110.6
O1—Cd1—N1^i^	137.9 (2)	C3—C4—H4A	110.6
O1^i^—Cd1—N1^i^	89.1 (2)	C5—C4—H4B	110.6
O1—Cd1—N1	89.1 (2)	C3—C4—H4B	110.6
O1^i^—Cd1—N1	137.9 (2)	H4A—C4—H4B	108.7
N1^i^—Cd1—N1	86.0 (3)	C1^ii^—C5—C4	117.8 (11)
O1—Cd1—O2^i^	106.12 (19)	C1^ii^—C5—C6	118.0 (11)
O1^i^—Cd1—O2^i^	55.22 (17)	C4—C5—C6	107.5 (11)
N1^i^—Cd1—O2^i^	115.6 (2)	C1^ii^—C5—H5	103.8
N1—Cd1—O2^i^	89.9 (2)	C4—C5—H5	103.8
O1—Cd1—O2	55.22 (17)	C6—C5—H5	103.8
O1^i^—Cd1—O2	106.12 (19)	C8—C6—C7	97.0 (16)
N1^i^—Cd1—O2	89.9 (2)	C8—C6—C5	114 (2)
N1—Cd1—O2	115.6 (2)	C7—C6—C5	114.2 (11)
O2^i^—Cd1—O2	145.7 (3)	C8—C6—C2	114.1 (19)
O1—Cd1—C1^i^	116.12 (19)	C7—C6—C2	114.7 (11)
O1^i^—Cd1—C1^i^	27.62 (17)	C5—C6—C2	103.2 (7)
N1^i^—Cd1—C1^i^	103.72 (19)	C6—C7—H7A	109.5
N1—Cd1—C1^i^	114.8 (2)	C6—C7—H7B	109.5
O2^i^—Cd1—C1^i^	27.59 (17)	H7A—C7—H7B	109.5
O2—Cd1—C1^i^	128.4 (2)	C6—C7—H7C	109.5
C1—O1—Cd1	93.7 (4)	H7A—C7—H7C	109.5
C1—O2—Cd1	91.2 (4)	H7B—C7—H7C	109.5
C13—N1—C9	115.7 (6)	C6—C8—H8A	109.5
C13—N1—Cd1	124.5 (5)	C6—C8—H8B	109.5
C9—N1—Cd1	119.6 (4)	H8A—C8—H8B	109.5
O2—C1—O1	119.9 (5)	C6—C8—H8C	109.5
O2—C1—C5^ii^	122.3 (8)	H8A—C8—H8C	109.5
O1—C1—C5^ii^	117.8 (8)	H8B—C8—H8C	109.5
O2—C1—C2	119.4 (7)	N1—C9—C10	125.5 (7)
O1—C1—C2	120.7 (7)	N1—C9—H9	117.2
C3—C2—C1	116.4 (10)	C10—C9—H9	117.2
C3—C2—C6	105.3 (9)	C11—C10—C9	118.3 (7)
C1—C2—C6	113.7 (10)	C11—C10—H10	120.9
C3—C2—H2	107.0	C9—C10—H10	120.9
C1—C2—H2	107.0	C12—C11—C10	116.2 (6)
C6—C2—H2	107.0	C12—C11—C11^iii^	122.8 (7)
C2—C3—C4	106.8 (10)	C10—C11—C11^iii^	121.0 (7)
C2—C3—H3A	110.4	C11—C12—C13	121.3 (6)
C4—C3—H3A	110.4	C11—C12—H12	119.4
C2—C3—H3B	110.4	C13—C12—H12	119.4
C4—C3—H3B	110.4	N1—C13—C12	122.9 (7)
H3A—C3—H3B	108.6	N1—C13—H13	118.5
C5—C4—C3	105.9 (11)	C12—C13—H13	118.5
			
O1^i^—Cd1—O1—C1	88.7 (4)	C5^ii^—C1—C2—C3	−154 (14)
N1^i^—Cd1—O1—C1	−39.8 (6)	O2—C1—C2—C6	101.9 (11)
N1—Cd1—O1—C1	−122.8 (5)	O1—C1—C2—C6	−81.0 (12)
O2^i^—Cd1—O1—C1	147.5 (4)	C5^ii^—C1—C2—C6	−32 (13)
O2—Cd1—O1—C1	−0.3 (4)	C1—C2—C3—C4	153.2 (14)
C1^i^—Cd1—O1—C1	119.8 (4)	C6—C2—C3—C4	26.3 (19)
O1—Cd1—O2—C1	0.3 (4)	C2—C3—C4—C5	−8(2)
O1^i^—Cd1—O2—C1	−115.9 (4)	C3—C4—C5—C1^ii^	−149.5 (17)
N1^i^—Cd1—O2—C1	155.1 (4)	C3—C4—C5—C6	−13 (2)
N1—Cd1—O2—C1	69.6 (5)	C1^ii^—C5—C6—C8	41 (2)
O2^i^—Cd1—O2—C1	−64.9 (4)	C4—C5—C6—C8	−95 (2)
C1^i^—Cd1—O2—C1	−97.3 (5)	C1^ii^—C5—C6—C7	−70 (2)
O1—Cd1—N1—C13	15.5 (6)	C4—C5—C6—C7	154.3 (15)
O1^i^—Cd1—N1—C13	153.2 (6)	C1^ii^—C5—C6—C2	165.3 (14)
N1^i^—Cd1—N1—C13	−122.6 (7)	C4—C5—C6—C2	29.1 (17)
O2^i^—Cd1—N1—C13	121.7 (6)	C3—C2—C6—C8	91 (2)
O2—Cd1—N1—C13	−34.6 (7)	C1—C2—C6—C8	−38.0 (19)
C1^i^—Cd1—N1—C13	134.1 (6)	C3—C2—C6—C7	−158.8 (13)
O1—Cd1—N1—C9	−170.5 (7)	C1—C2—C6—C7	72.7 (16)
O1^i^—Cd1—N1—C9	−32.9 (8)	C3—C2—C6—C5	−33.8 (14)
N1^i^—Cd1—N1—C9	51.3 (7)	C1—C2—C6—C5	−162.4 (12)
O2^i^—Cd1—N1—C9	−64.4 (7)	C13—N1—C9—C10	1.7 (15)
O2—Cd1—N1—C9	139.3 (7)	Cd1—N1—C9—C10	−172.8 (9)
C1^i^—Cd1—N1—C9	−52.0 (8)	N1—C9—C10—C11	0.4 (17)
Cd1—O2—C1—O1	−0.6 (7)	C9—C10—C11—C12	−2.6 (13)
Cd1—O2—C1—C5^ii^	−179.7 (11)	C9—C10—C11—C11^iii^	178.3 (9)
Cd1—O2—C1—C2	176.6 (7)	C10—C11—C12—C13	2.7 (12)
Cd1—O1—C1—O2	0.6 (7)	C11^iii^—C11—C12—C13	−178.2 (8)
Cd1—O1—C1—C5^ii^	179.8 (10)	C9—N1—C13—C12	−1.5 (12)
Cd1—O1—C1—C2	−176.5 (7)	Cd1—N1—C13—C12	172.6 (6)
O2—C1—C2—C3	−20.7 (16)	C11—C12—C13—N1	−0.7 (13)
O1—C1—C2—C3	156.4 (11)		

Symmetry codes: (i) −*x*+1, *y*, −*z*+1/2; (ii) −*x*, *y*, −*z*+1/2; (iii) −*x*+1, −*y*, −*z*+1.

## References

[bb1] Adhya, R. N., Ghosh, A. C. & Bardhan, J. C. (1956). *J. Chem. Soc.* pp. 358–361.

[bb2] Barbour, L. J. (2001). *J. Supramol. Chem.* **1**, 189–191.

[bb3] Camps, P. & Jaime, C. (1981). *Can. J. Chem.* **59**, 2848–2852.

[bb4] Higashi, T. (1995). *ABSCOR* Rigaku Corporation, Tokyo, Japan.

[bb5] Rigaku (1998). *RAPID-AUTO* Rigaku Corporation, Tokyo, Japan.

[bb6] Rigaku/MSC (2002). *CrystalClear* Rigaku/MSC Inc., The Woodlands, Texas, USA.

[bb7] Sheldrick, G. M. (2008). *Acta Cryst.* A**64**, 112–122. 10.1107/S0108767307043930 18156677

[bb8] Westrip, S. P. (2010). *J. Appl. Cryst.* **43**, 920–925.

